# Disease Burden from Hepatitis B Virus Infection in Guangdong Province, China

**DOI:** 10.3390/ijerph121114055

**Published:** 2015-11-02

**Authors:** Jianpeng Xiao, Hualiang Lin, Tao Liu, Weilin Zeng, Xing Li, Xiaoping Shao, Qiu Tan, Yanjun Xu, Xiaojun Xu, Huizhen Zheng, Wenjun Ma

**Affiliations:** 1 Guangdong Provincial Institute of Public Health, Guangdong Provincial Center for Disease Control and Prevention, Guangzhou 511430, China; E-Mails: jpengx@163.com (J.X.); hualianglin@gmail.com (H.L.); gztt_2002@163.com (T.L.); letitiazeng@foxmail.com (W.Z.); lixing.echo@foxmail.com (X.L.); 2 Institute of Immunization Program, Guangdong Provincial Center for Disease Control and Prevention, Guangzhou 511430, China; E-Mails: shaoxping@tom.com (X.S.); tq_1020@163.com (Q.T.); 3 Institute of Chronic Non-communicable Disease Control and Prevention, Guangdong Provincial Center for Disease Control and Prevention, Guangzhou 511430, China; E-Mails: gdxyj05@21cn.com (Y.X.); xu-yd@163.com (X.X.)

**Keywords:** hepatitis B virus, DALYs, burden of disease

## Abstract

*Objective*: To estimate the disease burden and financial burden attributed to hepatitis B virus (HBV) infection in Guangdong Province. *Methods*: Based on the data of incidence, mortality and healthcare cost of HBV-related diseases and other socio-economic data in Guangdong Province, we estimated deaths, disability-adjusted life-years (DALYs) and economic cost for the three HBV-related diseases—hepatitis B, liver cirrhosis and liver cancer—in Guangdong following the procedures developed for the global burden of disease study. Then disease burden and economic cost attributed to HBV infection was estimated. *Results*: HBV infection was estimated to have caused 33,600 (95% confidence interval (CI): 29,300–37,800) premature deaths and the loss of 583,200 (95% CI: 495,200–671,100) DALYs in Guangdong in 2005. The greatest loss of deaths and DALYs were from liver cancer. The 45–59 years age group had the greatest burden attributable to HBV infection. The estimated total annual cost of HBV-related diseases in Guangdong was RMB 10.8 (95% CI: 8.7–13.0) billion，the direct and indirect cost were RMB 2.6 (95% CI: 2.1–3.2) and 8.2 (95% CI: 6.6–9.8) billion. *Conclusions*: HBV infection is a great medical challenge as well as a significant economic burden to Guangdong Province. The results suggest that substantial health benefits could be gained by extending effective public health and clinical interventions to reduce HBV infection in Guangdong Province.

## 1. Instruction

Hepatitis B virus (HBV) infection is one of the most common and serious infectious diseases, affecting more than 2 billion people worldwide [1,2]. People with hepatitis B are at increased risk of developing hepatic decompensation, liver cirrhosis and liver cancer [3]. A recent estimation showed that HBV infection was the tenth leading cause of death and accounted for 786,000 deaths each year around the world [4,5]. 

Endemic HBV infection is a particularly serious public health problem in China [6,7]. There are still about 97 million HBV carriers, at least 20 million of them suffer from HBV infection alone or in combination with liver cirrhosis and/or liver cancer [6,8]. According to the Global Burden of Disease report, about 256,000 deaths could be attributed to HBV infection in China in 2010 [7].

Guangdong Province, located in south China, is the most populous province in China, with a population of about 104 million in 2010. The prevalence of HBV infection is one of the most serious problems in Guangdong. It was reported that 17.9% of general population and 19.9% of children were HBV carriers in Guangdong in 1992, ranking the first in China [9]. After that, a long-term program to vaccinate newborns against HBV was started in Guangdong. Although HBV prevalence had been reduced to 11.1% in the general population and 4.9% in the children by 2006 [9], a large part of adults still suffer from HBV infection. It is estimated that at least 10 million persons are carrying HBV in Guangdong, and 90% of them are adults [9]. In the next few decades HBV infection will highlight social issues in Guangdong. Facing the challenge of HBV infection and the limited health resources, a reasonable estimation of disease burden and efficient use of health resources are warranted. 

Recently, a few studies have reported the disease burden of HBV infection around the world [10,11,12,13,14,15]. For instance, a mathematical model estimated that about 620,000 persons died from HBV-related diseases worldwide [14]. A report estimated that the loss of 476,000 disability-adjusted life-years (DALYs) could be attributed to the HBV infection in Shandong Province, China [16]. Yang reported that financial costs of HBV-related diseases in South Korea was equivalent to 0.24% of the 2005 national GDP [15]. A study in Singapore showed that total direct cost of HBV infection was equivalent to 12% of the national healthcare expenditure [13]. Recently a project examined the medical costs associated with the management of chronic hepatitis B in a few areas in China [11,17]. However, limited data exist on the population-base burden of HBV infection in the whole Guangdong Province, or the corresponding financial burden.

Quantifying the disease burden caused by HBV infection is an important guide for policy development, priority setting and management of public health management. The aim of this study was therefore to assess the disease burden of HBV-related diseases including hepatitis B, liver cirrhosis and liver cancer, and estimate their financial burden to the society.

## 2. Materials and Methods

### 2.1. Data Collection

The incidence data of hepatitis B in 2005 in Guangdong were obtained from the Guangdong Provincial Center for Disease Control and Prevention. The incidence data was collected from 21 surveillance sites in Guangdong Province, which would represent the levels of the whole province [18]. 

Cause-specific mortality data of hepatitis B, liver cirrhosis and liver cancer were derived from the third retrospective mortality survey in Guangdong in 2006 [19]. About 15.2 million people, accounting for 10.1% of the total provincial population was investigated, which had been assessed and would well represent the situation of the whole province [19]. This survey was the largest and most comprehensive mortality survey in Guangdong in the past 10 years. The population data used in the study was collected from the Guangdong Population Statistical Yearbook. The cost data of HBV-related diseases were collected mainly from investigations conducted in Guangdong Province [20]. The investigations were conducted by collecting health care information, which reported the average direct cost for patients with HBV-related diseases in Guangdong Province. Gross national product (GNP) data were obtained from the Economy and Society Development Statistical Bulletin of Guangdong Province.

### 2.2. Statistical Analysis

Based on the data from numerous sources, we developed a plan to estimate the disease burden for hepatitis B, liver cirrhosis and liver cancer in Guangdong following the procedures developed for the Global Burden of Disease study [21,22]. We first calculated the death and DALYs of hepatitis B, liver cirrhosis and liver cancer in Guangdong, respectively, then estimated the disease burden and financial loss of the three diseases caused by HBV infection.

#### 2.2.1. DALYs Calculation

DALYs were calculated using the expression DALY = YLL + YLD, where YLL is years of life lost, and YLD is years lived with disability [21,22]. Specifically, premature death is calculated by YLL = N × L, where N = number of deaths due to condition, L = standard life expectancy at age of death (expectancy—age at death). The reference life expectancies at birth were 80.0 years for males and 82.5 years for women, based on the report of World Health Organization (WHO). YLD is determined by the number of years disabled weighed by the level of disability caused by a disability or disease using the formula YLD = I × DW × L. In this formula I = number of incident cases in the population, DW = disability weight of specific condition, and L = average duration of the case until remission or death (years). In the studies future years were also discounted at a 3% rate to account for future health care losses. The age weights (standard age weights = 0.04) implied the value of life depends on age was used in the estimation[23]. Disability weights were used according to the WHO reports (available at http://www.who.int/entity/healthinfo/bodreference disability weights.xls).

#### 2.2.2. DALYs for Hepatitis B 

YLLs were computed by multiplying the number of deaths due to acute and chronic hepatitis B by a reference life expectancy of different age groups and genders. For YLDs, the incidence, disability weight and duration of hepatitis B were used. Due to a lack of accurate data of disability weight and disease duration, the value of average age of death minus average age of onset was used to represent the average duration. The age-specific disability weights of hepatitis B were used according to the WHO reports; the disability weight coefficients of the 0–4, 5–14, 15+ year age groups were 0.17, 0.18 and 0.21, respectively. DALYs are calculated by taking the sum of YLLs and YLLs, then we estimated the DALYs in loss per 100,000 per year.

#### 2.2.3. DALYs for Liver Cirrhosis 

YLLs of liver cirrhosis were calculated by multiplying the number of deaths of liver cirrhosis by a reference life expectancy of different age groups and genders. Due to the limited data of liver cirrhosis incidence and prevalence in Guangdong, we used an indirect method to accordingly estimate the YLDs of liver cirrhosis [23]. In brief, we first collected the data of disease burden of China in 2008 on the WHO website (available in http://www.who.int/gho/mortality_burden_disease/en), then the YLDs to YLLs rate method was used to calculate YLDs by different age groups and genders for liver cirrhosis in Guangdong. 

#### 2.2.4. DALYs for Liver Cancer 

YLLs of liver cancer were calculated by multiplying the number of deaths of liver cancer by a reference life expectancy of different age groups and genders. YLDs of liver cancer were calculated using mortality, disability weight and duration of liver cancer. The incidence and prevalence data of liver cancer in Guangdong is limited. A previous study reported that liver cancer has a poor prognosis and its mortality was very close to its incidence in China, so we used mortality data to estimate the YLDs. Disease duration was defined as 1 years following a previous study [16]. The disability weight coefficients of was defined as 0.42 according to the WHO report (available in http://www.who.int/entity/healthinfo/bodreference disability weights.xls.).

### 2.3. Attributable Burden Estimation

As the information of medical history was incomplete, when estimating attributable deaths and DALYs of liver cirrhosis and liver cancer to HBV infection, we used the attributable fraction on the basis of previous reports [3,24,25,26]. In brief, we used attributable fractions of 40%–60% and 70%–90% for liver cirrhosis and liver cancer, respectively. 

### 2.4. Economic Burden Estimation

The economic burden of disease could include direct and indirect economic costs. The direct cost means the direct medical expenditures incurred by the health system to diagnose and treat health problems. The indirect cost implies the economic burden loss due to the illness or death [13]. In the study we roughly estimated direct expenditures and indirect costs of hepatitis B, liver cirrhosis and liver cancer based on available data sources. For direct expenditures, we collected the individual cost data of HBV-related diseases from recent investigations in Guangdong [10,20]. The average costs per person for hepatitis B, liver cirrhosis and liver cancer were estimated, respectively. The direct cost equal the estimated number of patients multiplied by the average cost. For indirect cost, the human capital method combined DALYs was adopted with the formula as: indirect economic burden = DALYs × GNP per person × productivity weight [27]. The GNP per person in Guangdong in 2005 was 21,701 RMB. The productivity weights of age groups of 0–14, 15–44, 45–59, and 60+ years were 0.15, 0.75, 0.80 and 0.1, respectively [27]. 

### 2.5. Uncertainty Analysis

To check the robustness of our estimates, uncertainty analyses were conducted by utilizing various assumptions regarding the estimates of incidence, mortality, life expectancy, disability weights and other variables. For instance, we used the incidence of hepatitis B from surveillance system of infectious disease, changed life expectancy based on the resent reports (the life expectancy at birth is 86.0 years), and used other disability weights, medical costs and attributable fractions from other reports.

## 3. Results

The incidence and mortality of hepatitis B in Guangdong Province in 2005 were 39.4 and 2.9 per 100,000, respectively (Table 1). Average age of hepatitis B onset was 45.2 years, and average age of death was 59.3 years, thus the average duration of hepatitis B was 14.1 years. Medical expenses on hepatitis B was 20,450 RMB per person [20]. The mortality of liver cirrhosis was 8.0 per 100,000, and about 50% (40%–60%) of the deaths may attribute to hepatitis B infection [3]. The Medical expense on liver cirrhosis was 42467 RMB per person [20]. The mortality of liver cancer was 37.0 per 100,000, and about 80% (70%–90%) of the deaths may attribute to hepatitis B infection [25,26]. The Medical expense on liver cirrhosis was 57692 RMB per person [20]. 

**Table 1:** Mortality of hepatitis B, liver cirrhosis and liver cancer in Guangdong Province and related parameters used in the study.

Variables	Incidence (Per 100000）	Mortality (Per 100000）	Disability Weight	Duration (Years)	Attributable Fraction (95% CI)	Medical Expense Per Capita (RMB)
**Hepatitis B**	39.4 (39.0–39.8)	2.9 (2.8–3.0)	0.21	14.1	1.0	20,450
Male	50.9 (50.2–51.6)	4.3 (4.1–4.5)	0.21	14.1	1.0	20,450
Female	27.0 (26.5–27.5)	1.5 (1.4–1.6)	0.21	14.1	1.0	20,450
**Liver cirrhosis**	−	8.0 (7.8–8.2)	−	−	0.5 (0.4–0.6)	42,467
Male	−	11.6 (11.3–11.9)	−	−	0.5 (0.4–0.6)	42,467
Female	−	4.4 (4.2–4.6)	−	−	0.5 (0.4–0.6)	42,467
**Liver cancer**	−	37.0 (36.6–37.4)	0.44	1.0	0.8 (0.7–0.9)	57,692
Male	−	56.1 (55.4–56.8)	0.42	1.0	0.8 (0.7–0.9)	57,692
Female	−	17.3 (16.9–17.7)	0.45	1.0	0.8 (0.7–0.9)	57,692

**Figure 1:**
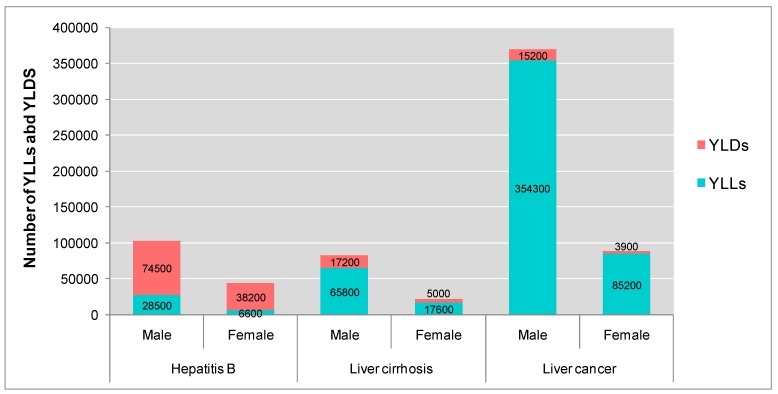
Number of YLLs and YLDs from hepatitis B, liver cirrhosis and liver cancer in Guangdong Province in 2005.

Figure 1 shows the DALYs loss from hepatitis B, liver cirrhosis and liver cancer in Guangdong Province in 2005. The three diseases led to a total of 712,000 DALYs lost in Guangdong in 2005. Liver cancer has the greatest disease burden, following by the liver cirrhosis and hepatitis B. YLDs was the main component in the disease burden of hepatitis B, while the YLLs was the major in disease burden of liver cirrhosis and liver cancer. Males had higher proportion of disease burden than females in all three diseases. 

Table 2 provides the attributable deaths and DALYs for the HBV-related diseases. HBV infection was estimated to have caused 33,600 (95% CI: 29,300–37,800) premature deaths and the loss of 583,200 (95% CI: 495,200–671,100) DALYs in Guangdong in 2005. The death rate is 36.5 per million and the DALY rate is 634.3 per million. Males had approximately 3.6 times more deaths attributable to HBV infection than females, with 25,600 deaths and 452,800 DALYs lost. The largest number of estimated deaths attributed to HBV infection was caused by liver cancer, namely 27,200 (95% CI: 23,800–30,600), including 20,900 in men and 6300 in women. The next was liver cirrhosis (3700 deaths) and hepatitis B (2700 deaths). In term of DALYs, the greatest loss was also from liver cancer with 382,600 (95% CI: 334,800–430,400) DALYs lost, followed by those from hepatitis B (147,800 DALYs) and liver cirrhosis (52,800 DALYs).

**Table 2:** Number of deaths and DALYs from HBV infection in Guangdong province.

Variables	Death (Thousands)	Death rate (Per 100,000)	DALYs (Thousands)	DALYs Rate (Per 100,000)
**Overall**	33.6	36.5	583.2	634.3
	(29.3–37.8)	(31.9–41.1)	(495.2–671.1)	(538.7–730.0)
Male	25.6	55.0	452.8	972.5
	(22.4–28.8)	(48–61.9)	(385.4–520.2)	(827.6–1117.3)
Female	8.0	17.5	130.4	287.3
	(6.9–9.0)	(15.3–19.8)	(109.9–150.9)	(242.1–332.5)
**Hepatitis B**	2.7	2.9	147.8	160.8
	(2.6–2.8)	(2.8–3.0)	(118.3–177.4)	(128.6–193.0)
Male	2.0	4.3	103.0	221.2
	(1.9–2.1)	(4.1–4.5)	(82.4–123.6)	(176.9–265.4)
Female	0.7	1.5	44.8	98.8
	(0.6–0.7)	(1.4–1.6)	(35.9–53.8)	(79.1–118.6)
**Liver cirrhosis**	3.7	4.0	52.8	57.4
	(2.9–4.4)	(3.2–4.8)	(42.2–63.3)	(45.9–68.9)
Male	2.7	5.8	41.5	89.1
	(2.2–3.2)	(4.6–7.0)	(33.2–49.8)	(71.3–106.9)
Female	1.0	2.2	11.3	24.8
	(0.8–1.2)	(1.8–2.6)	(9.0–13.5)	(19.8–29.8)
**Liver cancer**	27.2	29.6	382.6	416.1
	(23.8–30.6)	(25.9–33.3)	(334.8–430.4)	(364.1–468.1)
Male	20.9	44.9	308.3	662.2
	(18.3–23.5)	(39.3–50.5)	(269.8–346.9)	(579.4–745.0)
Female	6.3	13.8	74.3	163.6
	(5.5–7.1)	(12.1–15.6)	(65.0–83.5)	(143.2–184.1)

**Figure 2:**
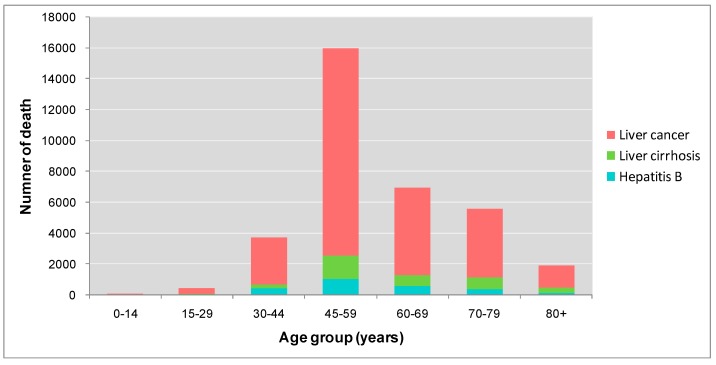
Number of deaths attributed to HBV infection in Guangdong Province in 2005, according to age group.

**Figure 3:**
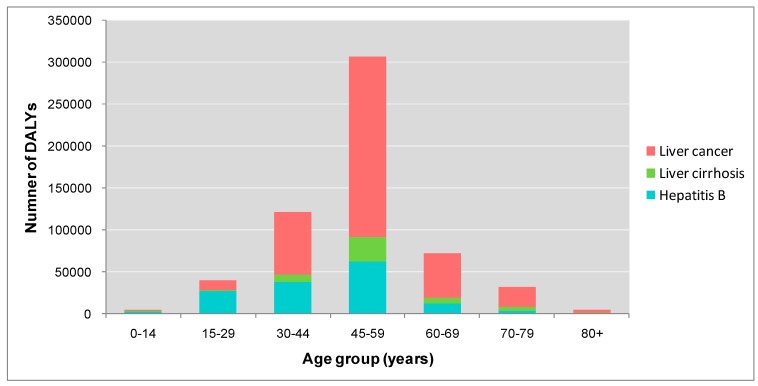
Number of DALYs attributed to HBV infection in Guangdong province in 2005, according to age group.

Figure 2 and Figure 3 show the numbers of deaths and DALYs from HBV infection by age group. The largest number of estimated deaths attributable to HBV infection was in the 45–59 years of age group, with approximately 15,900 deaths, including 13,400 due to liver cancer, 1500 from liver cirrhosis and 1000 from hepatitis B. The second largest number of deaths was found in the 60–69 year group, with 6900 deaths attributable to HBV infection. For DALYs, 45–59 years age group also have the greatest loss of DALYs of 306,100, including 214,500 in liver cancer, 62,300 in hepatitis B and 29,300 in liver cirrhosis. The second largest number of DALYs is found in 30–44 years group, with 121,400 DALYs loss attributing to HBV infection.

**Figure 4:**
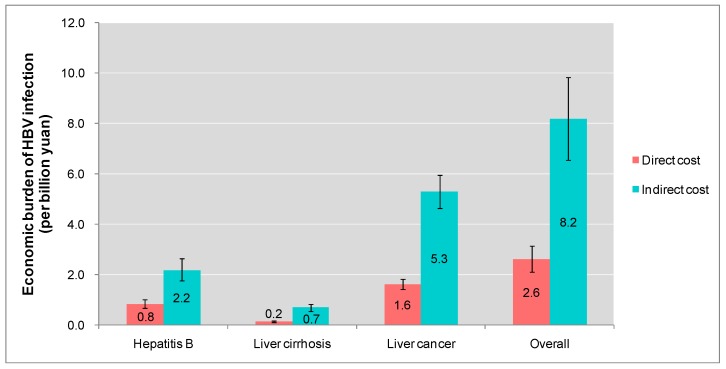
Estimation of direct and indirect economic cost from HBV infection in Guangdong province.

Figure 4 shows the economic burden from HBV infection in Guangdong province. It was estimated that HBV infection related disease may cost RMB 10.8 (95% CI: 8.7–13.0) billion, the direct and indirect cost were RMB 2.6 (95% CI: 2.1–3.2) and 8.2 (95% CI: 6.6–9.8) billion. 

About RMB 3.0 billion was expended on hepatitis B, of which indirect cost was RMB 0.8 billion. The economic burden of liver cirrhosis was RMB 0.9 billion, and the indirect cost was RMB 0.2 billion. The economic burden of liver cancer was RMB 6.9 billion, and the indirect economic burden was RMB 1.6 billion. 

We have performed a sensitivity analysis to estimate the effects of certain quantifiable uncertainties. Table 3 lists some assumptions underlying the estimates (column 1) and alternative conditions (column 2), and the numbers of deaths, DALYs or cost attributable to HBV have been recalculated accordingly (column 3). When altering different conditions, the estimation would take some changes. For instance, the pooled estimate of attributable fraction = 0.9 for liver cancer used would increase 3400 deaths.

**Table 3:** Sensitivity analysis of disease burden from HBV infection in Guangdong.

Assumption in Best Estimate	Alternative Condition	Effect on Number of Deaths, DALYs, Cost
1. Best estimate attributable fraction from meta analyses or original papers.	Upper 95% confidence interval	Death increased by 10.1% (3400 deaths)
	Lower 95% confidence interval	Death decreased by 10.1% (3400 deaths)
2. The cause-of-death statistics from the mortality survey reflect actual situation.	Using the mortality of 7 per thousand for Guangdong	Death increased by 6.1% (2097 deaths)
3. The life expectancy used can reflect actual situation in Guangdong.	New life table based on the 2010 GBD report was referenced	DALYs increased by 14.0% (95,648DALYs)
4. The incidence of hepatitis B used reflect actual situation in Guangdong.	Incidence of hepatitis B based on the surveillance system of infectious diseases	DALYs increased by 14.9% (101,800 DALYs)
5. The direct cost investigated from patients can represent provincial level.	Using the estimation from another survey in Guangdong	Direct cost decreased by 9% (RMB 0.2 billion)

## 4. Discussion

HBV infection is one of the most severe public health issues in Guangdong Province [6,28]. Our study provided a comprehensive assessment of the burden of HBV–related diseases in Guangdong. To the best of our knowledge, this study is the first report to assess the population-based burden from the HBV infection in Guangdong. We estimated that in 2005 HBV infection was responsible for about 33,600 premature deaths and 583,200 DALYs in Guangdong. The estimation of societal costs from HBV-related diseases was large too. We have estimated that HBV caused 33,600 deaths in 2005, corresponding to 5.7% of all deaths in Guangdong [19] and to about 12.0% of HBV-related deaths in China (about 256,000 deaths attributed to HBV infection in China each year) [7]. In addition, almost four-fifths of deaths and more than half of DALYs in Guangdong attributed to HBV were caused by liver cancer. Compared with the findings for Shandong Province in China [16], Guangdong has a larger portion of HBV-related deaths. This may be due to the higher prevalence of HBV in Guangdong than that in Shandong. Our findings indicate that the HBV infection situation in Guangdong is still pretty grim. Although Guangdong Province has successfully integrated hepatitis B vaccination into routine immunization programs and this has had a very significant impact on reducing the HBV carrier rate among children born after 1992 [9], there is still a large portion of adults suffering from HBV infection, especially in rural areas [6]. To further reduce the prevalence of HBV and the mortality of HBV infection in Guangdong, expanding vaccination coverage in adults and improving the treatment of the diseases is very essential.

Approximately three quarters of HBV-related mortality and DALYs occurred in men. The absolute number of deaths was higher in men than women for two reasons. First, the HBV-carrier rate was higher in men than women. It was reported that prevalence rate of HBsAg were 18.8% in men and 14.8% women [8]. Second, men infected with HBV are more likely to develop a chronic form of the disease [2]. For instance, the death rate of liver cancer was 56.1 per million for males and 17.3 per million for females in Guangdong in 2005 [19]. This finding adds another piece of evidence showing that hepatitis B hits men harder than women. In addition, we found the largest death and DALYs loss were observed in the 44–59 years of age group. Immunization programs would eventually lead to a disease reduction, but the largest disease burden was in the 44–59 years old group, so several decades will need to pass before the effect of vaccination can substantially reduce the burden [7]. 

We found that the chronic hepatitis B infection and its associated complications are a significant economic burden on the Guangdong population. The estimated total annual cost of HBV-related diseases in Guangdong was RMB 10.8 billion. Based on the estimation, the total direct cost was equivalent to 3.2% of healthcare expenditures in Guangdong (Guangdong spent about RMB 82.4 billion or 0.37% of GDP on healthcare in 2005). In contrast with some reports [3,13,15], we found that the highest direct cost contributor was liver cancer and not hepatitis B. This may be because Guangdong Province has a larger proportion of HBV-related liver cancer than other regions, as liver cancer is always the top killer in Guangdong Province [19]. In addition, indirect costs represented 75.9% (8.2/10.8) of the total cost of HBV infection in Guangdong, which highlights the substantial financial burden borne by HBV-infected patients. Above all, it is evident that HBV-related diseases represent a significant cost burden to the Guangdong health care system. The prevention or delay of chronic hepatitis B liver disease progression in Guangdong could result in substantial economic benefits to the whole society. 

Our study had a few limitations. First, due to data availability, YLDs for liver cirrhosis and liver cancer were estimated using the indirect method. Due to the variation of social economy and health systems in different regions, the estimated DALYs may not be perfectly accurate. Second, because the information is incomplete, we did not perform a separate calculation for acute and chronic hepatitis B, and only estimated the total burden of hepatitis B. Third, the attributable fraction of HBV infection used in the present study was based on other reports of China, that may not reflect the real situation in Guangdong. Fourth, due to the limitations of a sampling survey, the average direct cost for the three HBV-relative diseases may not represent well the real situation at a provincial level. Finally, the mortality data used in the study was collected in 2006. Making it relatively old, while the mortality survey is the latest and largest and was performed recently. As the HBV infection and mortality patterns have changed during the past decade, further study using updated data would be needed.

Nevertheless, it is evident that HBV-related diseases are a significant burden to the health care system. Over the past 20 years, Guangdong has made significant efforts to shed its “leader in HVB infection” title by investing large amounts of money on vaccination, and the treatment of liver diseases. However, many challenges remain that must be tackled collaboratively [6]. HBV immunization is a most useful program with substantial health promotion and economic benefits to the whole of society.

## 5. Conclusions

HBV infection is a great medical challenge as well as a significant financial burden in Guangdong Province. This emphasizes the importance of the prevention and treatment of HBV-related diseases from the perspective of Guangdong society.
